# Assessing Children’s Executive Function: BADS-C Validity

**DOI:** 10.3389/fpsyg.2021.626291

**Published:** 2021-02-19

**Authors:** Jessica Fish, F. Colin Wilson

**Affiliations:** ^1^Institute of Health and Wellbeing, College of Medical, Veterinary and Life Sciences, University of Glasgow, Glasgow, United Kingdom; ^2^Regional Acquired Brain Injury Unit, Musgrave Park Hospital, Belfast Health and Social Care Trust, Belfast, United Kingdom

**Keywords:** executive function, assessment, ecological validity, child development, cognitive function

## Abstract

**Objectives:**

To investigate the external and ecological validity of a standardized test of children’s executive functioning (EF), the Behavioral Assessment of the Dysexecutive Syndrome for Children (BADS-C).

**Background:**

There are few standardized measures for assessing executive functions in children, and the evidence for the validity of most measures is currently limited.

**Method:**

A normative sample of 256 children and adolescents from age 8–16 years completed the BADS-C, and a parent or teacher completed rating scales of the child’s everyday problems related to EF (Children’s version of the Dysexecutive Questionnaire; DEX-C) and Strengths and Difficulties Questionnaire (SDQ), a commonly used measure of emotional, social, cognitive, and behavioral problems.

**Results:**

Exploratory factor analyses yielded a two-factor structure to the BADS-C, indicative of monitoring and abstract reasoning processes, and a three-factor structure to the DEX-C, reflecting behavioral, and cognitive components of the dysexecutive syndrome as well as emotional responsiveness. Regression analyses showed significant relationships between BADS-C scores and everyday functioning as reported on the DEX and SDQ. Furthermore, there were significant differences in BADS-C scores between those children in the upper and lower quartiles on the SDQ.

**Conclusion:**

Results provide tentative evidence of BADS-C and DEX-C construct, convergent and predictive validity.

## Introduction

The term “executive functioning” (EF) refers to the processes involved in the coordination of more basic cognitive functions, and hence produce organized, goal-directed, behavior (Welsh and Pennington, 1988; Alvarez and Emory, 2006). It is an overarching term which includes cognitive functions such as planning, problem solving, fluency, attentional control, working memory, inhibition as well as cognitive flexibility (Nyongesa et al., 2019) although precise consensus remains elusive. Notwithstanding EF has an important role in mediating the development of socio-emotional and educational attainments (Riggs et al., 2006) and is known to be a predictor of future life outcomes including productivity (Diamond, 2013). Impairments of EF have been reported in many pediatric clinical groups including Attention Deficit/Hyperactivity Disorder (Barkley, 1997; Willcutt et al., 2005), Pervasive Developmental Disorders such as Autism (Hill, 2004; Robinson et al., 2009), Syndromes associated with Intellectual Disability such as Fragile X (Cornish et al., 2004), and Down’s Syndrome (Lanfranchi et al., 2010), Epilepsy (Parrish et al., 2007) as well as Traumatic and other Acquired Brain Injuries (Slomine et al., 2002; Chevignard et al., 2010). Such impairments can have detrimental effects on behavior, social development and educational outcomes (Biederman et al., 2004; Tonks et al., 2011), and consequently the identification of problems with EF is of great clinical importance.

The most influential models of EF are those derived from adult neuropsychology, specifically studies of patients with circumscribed lesions to areas of prefrontal cortex. It is known that such patients can display a variety of striking changes in cognition, behavior and perceived “personality.” Stuss and Benson (1984) for example, specified six common features as: the separation of action from knowledge, difficulty in sequencing language and behavior, problems in forming and shifting cognitive “set,” reduced resistance to interference, impaired monitoring of behavior, and an acquired attitude of unconcern, unawareness or apathy. Baddeley and Wilson (1988) employed the term “dysexecutive syndrome” to represent this cluster of impairments, due to the role of the central executive component of working memory in this pattern of behavior. This model, along with a number of other related theoretical frameworks such the Global Workspace Model (Dehaene et al., 1998; Dehaene and Naccache, 2001) and the Contention Scheduling and Supervisory Attention Systems proposed by Norman and Shallice (1986) and Shallice and Burgess (1996), posit a form of competition between concurrently active goals that, within a limited-capacity system, serves to organize behavior. For many tasks, the relative strengths of environmental triggers for routine actions, and the value of the expected reward from an action, may be quite sufficient to produce coherent behavior in a relatively “automatic” fashion. A second level of general (as opposed to modality- or task-specific) control is also suggested, which is associated with conscious or effortful processing and that can endogenously adjust the “weight” of competing goals in accordance with environmental and internal factors. This is particularly associated with the function of a prefrontal or fronto-parietal network (Duncan, 2006). Data from human and animal lesion studies, as well as structural and functional brain imaging, converge on this matter at the broadest level (Milner, 1982; D’Esposito et al., 1995; Shallice and Burgess, 1996), though the specific roles of sub-regions of prefrontal cortex remains the subject of intense debate (Duncan and Owen, 2000; Stuss, 2006).

Executive functioning measurement is notoriously difficult due to the inherent differences between everyday situations that tax executive functions and the context of a typical neuropsychological assessment. There are well-documented reports of individuals with striking functional impairments performing within the expected range on traditional neuropsychological tests (Shallice and Burgess, 1991). Factors thought to contribute toward this discrepancy include the novelty of associated tasks, the degree of structure afforded by the respective settings, the clarity of relevant goals, and presence of distractions to name but a few (Silver, 2000; Emslie et al., 2003). The Behavioral Assessment of the Dysexecutive Syndrome (BADS; Wilson et al., 1996) was developed for adults with the particular intention to provide an ecologically valid assessment that captures the more elusive aspects of the dysexecutive syndrome frequently missed by traditional tests, whilst being informed by more contemporary neuropsychological models. It is now a widely-used test within the United Kingdom and there is good evidence for its validity (Burgess et al., 1998, 2006). The BADS battery detected significant differences between children with ADHD, those with Acquired Brain Injury and age and IQ matched participants (Hughes et al., 2013). Notwithstanding, Anderson (1998) critically reviewed EF measures in children and adolescents and observed the selection to be seriously lacking, with standardized batteries reported to neglect measures of EF, and with those tests available being of little interest to children, lacking appropriate normative data, and being difficult to interpret due to the involvement of lower-level cognitive skills that are themselves incompletely developed. Whilst this review is now somewhat dated, nevertheless the conclusions reached remain contemporary (Roy et al., 2015). In particular, Nyongesa et al. (2019) in a scoping review of 705 studies examining EF in adolescents from 2002–2017 observed that less than seven percent (*n* = 48) reported on the reliability and/or validity of EF measures employed which were limited to high income countries. This review underscored the importance of considering the psychometric properties of EF measures, given that the existing evidence remains limited.

With these above issues in mind, Emslie et al. (2003) developed the Behavioral Assessment of the Dysexecutive Syndrome for Children (BADS-C) to address the need for a reliable and valid assessment of executive functions that included child-friendly materials, standardized administration and scoring instructions as well as comprehensive norms. BADS-C battery consists of six subtests: the Playing Cards Test, the Water Test, the Key Search Test, Zoo Map Tests 1 and 2, and the Six Part Test. Emslie et al. (2003) reported that the battery has excellent inter-rater reliability for the majority of measures (0.91–1.0), with lower reliability (0.53) for only one measure, the number of perseverative errors on the Water test. Test re-test reliability was assessed after 3–4 weeks, and significant improvements in performance were found for the Playing Cards and Six Parts tests. In addition, all children obtained the maximum score on the Water test on second administration which is not unsurprising given the nature of the tasks (i.e., where novelty is a component) and short testing interval. Correlations between BADS-C scores and scores on the Strengths and Difficulties Questionnaire (SDQ) were also examined, with the pattern of results suggesting correspondence between behavioral performance and informant-rated problems in everyday life. Therefore, there is some evidence regarding the BADS-C validity. Engel-Yeger et al. (2009) employed BADS-C in a normative sample of over 200 Arab-Israeli children and adolescents (aged 8–15). This study did not observed significant gender, familial socio-economic status nor parental level of education differences on the BADS-C. Notwithstanding as expected older children and adolescents performed better than younger children. As an aside, Willner et al. (2010) utilized the BADS-C as well as Cambridge Executive Functioning Assessment (CEFA) to assess EF in forty adults with mild to moderate learning disability. This study found that BADS-C scores were much lower in their sample than those observed in the BADS-C normative sample. More recently, Roy et al. (2015) utilizing a French version of BADS-C in a group of 120 children (aged 7–12) showed age but not gender based developmental trajectories whilst simultaneously observing weak correlations between BADS-C scores, IQ and parental education. Accordingly, further BADS-C investigations might be useful to aid clinicians and researchers in the interpretation of their results within child and adolescent samples. Hence, this paper reports results from four relevant secondary analyses extracted from the BADS-C standardization sample. These analyses relate to the factor structure of the BADS-C and its accompanying questionnaire, the Dysexecutive Questionnaire for Children (DEX-C), which were intended to inform their construct validity; and analyses of the associations between scores on these measures and everyday difficulties as measured by the SDQ, and a comparison of the BADS-C performance of children showing everyday difficulties and those not showing such difficulties, which were intended to examine construct, convergent and to a lesser degree the predictive validity of the BADS-C.

## Materials and Methods

### Participants

All participants were recruited on the basis of multi-center ethics approval granted by Cambridge Local Research Ethics Committee (LREC). The majority of participants in this study were recruited from schools in the east of England (United Kingdom). Letters explaining the project and asking for consent to participate were sent to the parents of all pupils in the relevant age groups (8–16 years) at these schools. Positive responses ranged from 40 to 95 percent, being consistently high in the primary schools and declining with increasing age in the secondary schools. A further 30 or so children who had taken part in a previous, unrelated research project were recruited on an individual basis.

After approximately 230 children had been tested, the mean estimated IQ for each age group was calculated using the Basic Reading test of the Wechsler Objective Reading Dimensions test (WORD; Wechsler, 1993). The majority of participants fell into the “average” ability range, so to ensure the extremes were not under-represented, subsequent recruitment was targeted at specific age and ability levels. Head teachers of a further group of schools agreed to recruit on this basis. In total, 260 individuals were assessed, though data from four children falling within the intellectual disability range (estimated IQ of ≤70) were excluded. Suffice to state, access to the full data-set is available on request to the first author.

The final normative group comprised 256 children (114 males, 142 females) across eight age bands from 8 years 0 months and 15 years 11 months. Chi squared tests confirmed that there were no systematic differences in the number of participants in each age band [χ^2^_*df(*__7__)_ = 2.75, *p* = 0.91; range = 29–40], and that the numerical difference in the proportion of males to females was not statistically significant [χ^2^_*df(*__7__)_ = 3.06, *p* = 0.08]. The mean estimated IQ of the group as a whole was 100.5 (SD = 12.7), and a boxplot showed that scores were normally distributed. Univariate ANOVA showed that estimated IQ did not vary significantly as a function of age group [*F*(1,7) = 2.01, *p* = 0.055], or sex [*F*(1,1) = 0.098, *p* = 0.755], and nor was there an interaction [*F*(1,7) = 0.923, *p* = 0.489]. Table 1 provides further details of participants and estimated IQ according to age group and gender.

**TABLE 1:** Participants and estimated IQ by age and gender.

Age in years	Sex	*N*	Estimated IQ			
			Mean	SD	Minimum	Maximum
8	Female	24	102.67	14.156	72	126
	Male	16	104.56	12.431	82	122
	Total	40	103.43	13.361	72	126
9	Female	24	104.33	11.970	83	127
	Male	10	102.40	14.909	78	120
	Total	34	103.76	12.699	78	127
10	Female	16	105.87	8.374	90	122
	Male	15	97.73	11.640	78	120
	Total	31	101.94	10.742	78	122
11	Female	13	93.46	14.069	73	117
	Male	17	93.88	14.044	71	113
	Total	30	93.70	13.812	71	117
12	Female	17	97.94	12.487	78	118
	Male	14	100.57	16.018	71	122
	Total	31	99.13	14.004	71	122
13	Female	17	98.24	13.184	75	117
	Male	13	99.62	9.614	87	114
	Total	30	98.83	11.603	75	117
14	Female	15	97.93	12.092	81	117
	Male	14	102.86	10.257	85	118
	Total	29	100.31	11.324	81	118
15	Female	16	104.38	10.282	75	115
	Male	15	99.20	12.405	77	114
	Total	31	101.87	11.471	75	115
Total	Female	142	101.06	12.577	72	127
	Male	114	99.91	12.820	71	122
	Total	256	100.55	12.674	71	127

**FIGURE 1:**
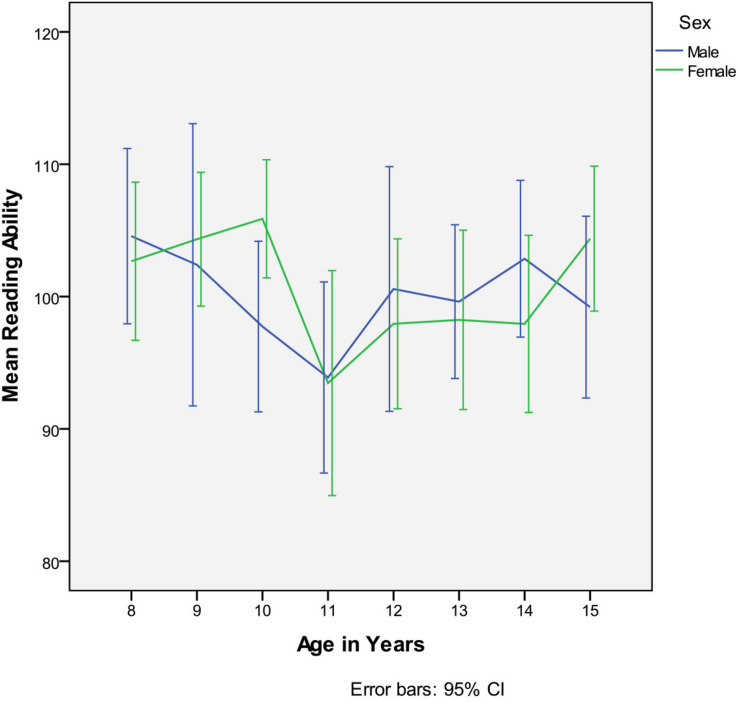
Estimated IQ by age group and sex.

ANOVA showed no evidence of a systematic variation in estimated IQ by age band or sex. Notwithstanding Figure 1 illustrates the 11-year old age group had a somewhat reduced mean IQ in relation to other age bands due to the presence of a greater proportion of children with estimated IQs below 90. However, the difference did not reach statistical significance and as all IQs were above the cut off which may indicate intellectual disability (estimated IQ of ≤70), no further cases were excluded from the analysis.

### Procedure

Each participant was test individually in a quiet room, either at their school or a research institution. Participants completed the BADS-C and the Basic Reading Test from the WORD (Wechsler, 1993). The teachers of all primary-school-age children and the parents of all secondary-school-age children and adolescents were asked to complete two questionnaires, the DEX-C (Emslie et al., 2003) and the SDQ (Goodman, 1997).

### Measures

#### The Behavioral Assessment of the Dysexecutive Syndrome for Children (Emslie et al., 2003)

The BADS-C consists of six subtests: the Playing Cards Test, the Water Test, the Key Search Test, Zoo Map Tests 1 and 2, and the Six Part Test. *The Playing Cards Test* requires children to establish and then update a pattern of responding. The child is shown a series of pictures of playing cards, and is asked to give verbal responses according to one “easy” rule (say “yes” to red and “no” to black), and then according to a new, more complex, rule (say “yes” if the card is the same color as the one before it, and “no” if it is not). This test constitutes a measure of cognitive flexibility. Scores are derived on the basis of time taken and uncorrected errors made.

The Water Test is a novel problem-solving task. Children are presented with an array of items and asked to find a solution to a practical problem according to a set of rules (to retrieve a cork from a tube using any item from the array including an empty container, a beaker of water with a plastic lid, and a wire hook, but without touching the lid with their fingers). A prompt detailing the first step (remove the lid with the wire) may be given if a child makes perseverative errors or has not completed the first step within 75 s. Scores are derived from the number of stages completed correctly, and the time taken.

The Key Search Test is a measure of planning ability that involves presenting the child with an A4 sheet of paper with a large square printed on it, that they are asked to imagine represents a large field in which they have lost a key. The child is asked to draw a line to show how they would go about finding the key. Scores are derived on the basis of overall quality of the plan, according to set criteria detailed in the manual.

The Zoo Map tests also measure planning. The child is shown a map of a zoo and asked, in version 1 of the test, to plan a route around it so that they may see a prescribed set of attractions, according to particular rules (e.g., not using particular walkways more than once, and with specified start and end points). Version 2 of the test is a measure of following instructions, the child is asked to follow a written plan of the route. Performance on the low-demand Part 2 facilitates interpretation of Part 1, in terms of excluding comprehension problems as a basis for mistakes on Part 1.

The Six Parts test is a multifaceted test of planning, scheduling and performance monitoring. Children are given three simple tasks to complete (sorting, simple arithmetic, and picture naming), each with two versions. They are given 5 min in which to attempt something from each task, according to the rule that they must not follow one version of a task with the other version of the same task. A clock and written summary of the rules is provided. Scores are derived from the number of tasks completed and rules broken.

In the published test, raw scores were converted to scaled scores. Scaled scores for all six subtests take into account the strong impact of age on test performance (as expected, there were significant linear trends for all raw scores). For all but two subtests, Key Search and Zoo Map 2, scaled scores also take into account estimated IQ within three bands, because estimated ability was found to have a moderate impact upon performance on these tasks. Further detail on the derivation of scaled scores can be found in the test manual (Emslie et al., 2003).

#### Basic Reading Test From WORD (Wechsler, 1993)

This brief test assesses decoding of letters and words, in younger and/or less able children, and progresses to reading aloud single words of increasing complexity. The average correlations between the BRT and verbal and full-scale IQ was calculated with a Fisher’s *Z* transformation on the *r*-values quoted in the WORD manual for each year group from 8 to 16 years. The average correlation between BRT and WISC-III verbal IQ was 0.57 and between BRT and WISC-III full-scale IQ was 0.52. This indicates that there is a reasonable association between BADS-C performance and general intellectual ability.

#### The Dysexecutive Questionnaire for Children (Emslie et al., 2003)

This questionnaire, which forms a supplement to the BADS-C battery, consists of 20 items related to cognitive, social and behavioral components of the dysexecutive syndrome, with each items rated on a 5-point Likert scale with the anchor points of “rarely” to “very often.” The DEX-C should be completed by someone who knows the child well, such as a parent, other family member, teacher or care worker. During BADS-C test development, teachers of primary school aged children completed DEX-C. As such teachers of primary school aged children spend significant more time with pupils than their high school equivalents. Whereas, for those in secondary level education, parents completed DEX-C. This process was also deployed for the second questionnaire measure.

#### The Strengths and Difficulties Questionnaire (Goodman, 1997)

This widely-used measure comprises 25 items, with 5 items in each of the following domains: emotional symptoms, conduct problems, hyperactivity/inattention, peer relationship problems, and pro-social behavior. There are versions for ratings of teachers and parents. Scores from the first four domains are grouped to form a “total difficulties score.” In this study, teacher ratings were obtained for children under 11 years, and parent ratings for children older than 11 years. Of note, Stone et al. (2010) who reviewed 48 studies among children from age 4–12 years observed satisfactory internal consistency, test-retest reliability and inter-rater agreement for both parent and teacher versions. Reliability for the teacher version was noted to be somewhat stronger than the parent version. Of the studies examining construct validity, most yielded a five-factor structure for the SDQ. However, more recently, Cheng et al. (2018) in a study involving children aged 6–11 years of age across seven European countries excluding United Kingdom observed low to moderate parent-teacher agreement across the five SDQ subscales and helpfully offered a number of explanatory factors to account for informant disagreement.

## Results

### Analysis I: Factor Structure of the BADS-C

The BADS and BADS-C are intended to encompass a variety of components of the “dysexecutive” syndrome, including planning, strategic behavior, time management, cognitive flexibility, and abstract thinking. It is therefore likely that the battery consists of latent subscales reflecting different EF domains. To investigate this issue scaled scores of the six BADS-C subtests were entered into a factor analysis with extraction by the Principal Components method and using Varimax rotation. Table 2 shows a two-factor solution with the first component having an Eigenvalue of 1.42 after rotation and explaining 24.8% of variance, and the second having an Eigenvalue of 1.14, explaining 19.1% of variance. The first component loaded on Playing Cards, Zoo Map 1, Zoo Map 2, and to a lesser degree the Six Parts Test, whereas the second loaded on Water and Key Search tests only. These components have been labeled “monitoring” versus “abstraction” tasks, respectively. The communalities for each variable were above0.3, with the exception of the Six Parts Test. This could be a result of the measure being unreliable, or it measuring something different than other variables. Given the extensive empirical evaluation of the measure from which the Six Parts test was derived (Wilson et al., 1996) the latter seems more likely. In any case, it is unsurprising that neither component loads particularly strongly on this variable.

**TABLE 2:** Factor loadings and communalities based on a principal components analysis with Varimax rotation for BADS-C subtests.

	1: Monitoring	2: Abstraction	Communalities
Playing cards	0.632	0.176	0.43
Water	–0.009	0.758	0.57
Key search	0.073	0.729	0.54
Zoo map 1	0.566	0.055	0.32
Zoo map 2	0.710	–0.016	0.51
Six parts	0.435	–0.053	0.19

### Analysis II: Factor Structure of the DEX-C

To investigate the latent structure of the DEX-C, all 20 questionnaire items were entered into a factor analysis with extraction by the Principal Components method using Varimax rotation. Communalities for these variable were all >0.5 indicating substantial shared variance. Table 3 illustrates the three-factor solution which explained 67% of the total variance. After rotation, the first component had an Eigenvalue of 7.3, explaining 36.5% of the variance, the second an Eigenvalue of 4.16, explaining 20.8% of variance, and the third of 2.04, explaining 10.2% of the variance. Before labeling the components, the correlation matrix was examined with only the strongest loading for each item remaining (i.e., one component for each item). The domains addressed by each DEX item were then added to the matrix to facilitate the identification of themes within each component, and the components were thus labeled “behavior,” “cognition,” and “responsiveness.” This does not follow the factor structure previously identified by Burgess et al. for the DEX questionnaire in adults (inhibition, intentionality, and executive memory), but is broadly consistent with Stuss and Benson’s (1984) delineation of emotional/personality, motivational, behavioral, and cognitive aspects of the dysexecutive syndrome which strongly influenced BADS-C development.

**TABLE 3:** Rotated Component Matrix for DEX-C items showing strongest loading per item.

Item	Content	Component			Communalities
		Behavior	Cognition	Responsiveness	
1	Abstract thinking problems		0.837		0.720
2	Impulsivity	0.701			0.649
3	Confabulation	0.651			0.619
4	Planning problems		0.831		0.791
5	Euphoria	0.800			0.688
6	Temporal sequencing problems		0.790		0.741
7	Lack of insight/social awareness		0.581		0.683
8	Apathy/lack of drive			0.808	0.743
9	Disinhibition	0.735			0.635
10	Variable motivation	0.729			0.648
11	Shallow affective responses			0.684	0.496
12	Aggression	0.675			0.667
13	Lack of concern	0.750			0.724
14	Perseveration	0.731			0.631
15	Restlessness-hyperkinesis	0.785			0.684
16	Inability to inhibit responses	0.826			0.773
17	Knowing-doing dissociation	0.698			0.693
18	Distractibility		0.605		0.661
19	Poor decision-making ability		0.657		0.582
20	No concern for social rules	0.701			0.659

### Analysis III: Relationship Between BADS-C Subtest Performance and Indices of Everyday Functioning

The BADS-C manual reported significant correlations between BADS-C total score and all indices from the SDQ problem-focussed sub-scales and these analyses are not repeated here. However, the relationships between the newly identified variables and reports of everyday function are presented in Table 4 below. From the table, there are moderate correlations between the DEX-C factors and SDQ subscales, and smaller but non-zero correlations between BADS-C factors and SDQ subscale and total scores (which are not trivial considering the difference in measuring child behavior directly versus obtaining informant ratings).

**TABLE 4:** Spearman’s Correlations between BADS-C and SDQ subscale scores, and between DEX-C factors and SDQ subscale scores.

		DEX-C Beh.	DEX-C Cog.	DEX-C Resp.	BADS-C monitor	BADS-C abstract	BADS-C six parts
Prosocial	R	−0.398**	−0.270**	−0.353**	0.067	0.067	0.066
	P	0.000	0.000	0.000	0.326	0.321	0.327
	N	219	219	219	220	220	220
Emotional symptoms	R	0.327**	0.315**	0.175**	−0.166*	−0.137*	−0.219**
	P	0.000	0.000	0.010	0.014	0.043	0.001
	N	217	217	217	218	218	218
Conduct Problems	R	0.647**	0.131	0.282**	–0.131	–0.080	−0.135*
	P	0.000	0.054	0.000	0.053	0.238	0.046
	N	217	217	217	218	218	218
Hyperactivity	R	0.626**	0.458**	0.069	−0.167*	–0.122	−0.176**
	P	0.000	0.000	0.312	0.014	0.073	0.010
	N	214	214	214	215	215	215
Peer Problems	R	0.327**	0.243**	0.273**	–0.110	−0.135*	−0.227**
	P	0.000	0.000	0.000	0.107	0.046	0.001
	N	216	216	216	217	217	217
Total Difficulties	R	0.623**	0.395**	0.244**	−0.183**	−0.155*	−0.236**
	P	0.000	0.000	0.000	0.006	0.021	0.000
	N	219	219	219	222	222	222

*^∗^refers to significant and ^∗∗^highly significant.*

To examine the value of the BADS-C subtests in predicting problems in everyday life, the six BADS-C subtest scaled scores were entered into a stepwise multiple regression on the dependent variable of SDQ total difficulties score. A model based on the Six Parts and Key Search scores was found to predict everyday problems, however, the model accounted for only 8% of the variance [*R*^2 adj^ = 0.08, *F*(2,221) = 10.40, and *p* < 0.001; Six Parts β = -0.229, *p* < 0.001; and Key Search β = -0.176, *p* = 0.007]. Repeating this analysis using the Factor Analysis-derived scores for Abstraction, Monitoring, and Six Parts tests resulted in another significant model consisting of Abstraction and Six Parts variables, which accounted for 7.4% of the variance [*R*^2 adj^ = 0.07, *F*(2,221) = 9.776, and *p* < 0.001; Six Parts β = -0.221, *p* < 0.001; and Abstraction β = -0.163, *p* = 0.013].

To investigate the power of BADS-C subscale scores to predict DEX-C scores, the above analyses was repeated on the dependent variable of DEX-C total scores. Likewise using the six subtest scores, a model based on the Six Parts and Key Search scores was significant but accounted for only 4% of the variance [*R*^2 adj^ = 0.04, *F*(2,222) = 6.08, and *p* = 0.003; Six Parts β = -0.158, *p* < 0.017; and Key Search β = -0.159, *p* = 0.016]. The equivalent analysis using the factor analysis-derived scores was significant only with the Six Parts score accounting for 2% of the variance [*R*^2 adj^ = 0.02, *F*(1,222) = 6.15, and *p* = 0.014; β = -0.165, *p* < 0.014].

In summary, these statistically significant regression models indicate that there is a robust relationship between BADS-C subtest scores and indices of everyday functioning, and yields evidence that the measure has construct validity. However, the small proportion of variance explained by each model indicates that BADS-C scores would not be particularly useful in predicting everyday problems within the general population. Nevertheless, these modest relationships are of interest given the control sample includes children exhibiting few if any EF difficulties all of whom are in receipt of normal state education provision.

### Analysis IV: Comparing the BADS-C Performance of Children With Low and High SDQ Scores

To further examine the relationship between BADS-C performance and everyday functioning, BADS-C scores of children falling in the lower and upper quartiles for SDQ total difficulties were compared. The median score on the SDQ total difficulties scale was 6 (mean 7.7, SD 6.6, range 0–34, 25th percentile = 3, and 75th percentile = 11). The “Low SDQ” group consequently comprised 72 children, and the “high SDQ” group included 62 children (the numbers are not equivalent as different numbers of children obtaining the criterion scores).

As Table 5 illustrates there was significant gender difference between the “low SDQ” and “high SDQ” groups (χ^2^ = 4.81, *p* = 0.037), with the low SDQ group containing a disproportionate number of girls. There was also a statistically significant 10-point difference in estimated IQ between the groups [*t*(132) = 5.22, *p* < 0.001].

**TABLE 5:** Gender and IQ distributions in groups obtaining low and high SDQ scores.

		N	Mean	SD	Min	Max
Low SDQ	Male	27	106.41	9.605	78	120
	Female	45	105.47	10.778	75	127
	*Total*	*72*	*105*.*82*	*10*.*295*	*75*	*127*
High SDQ	Male	35	95.60	12.816	71	121
	Female	27	94.52	14.273	72	126
	*Total*	*62*	*95*.*13*	*13*.*367*	*71*	*126*

**FIGURE 2:**
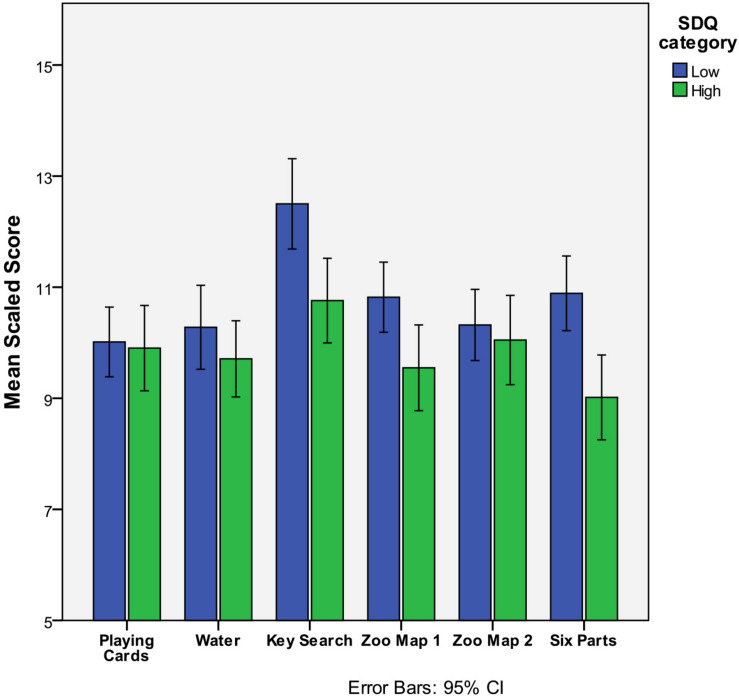
Bar chart of BADS-C subtest Scaled scores according to SDQ score categorization. The error bars indicate 95% Confidence Intervals, and illustrate significant differences in performance on Key Search, Zoo Map 1, and Six Parts tests only.

A MANOVA was therefore conducted on the BADS-C subtest scores by SDQ score group, with estimated IQ included as a covariate^1^. The multivariate effect was significant *F*(6,126) = 4.4, *p* < 0.001, ηp^2^ = 0.173, a large effect. Between-subjects effects for the six subtests revealed significant differences between high and low SDQ groups with a small-medium effect size for Key Search [*F*(1, 31) = 7.4, *p* = 0.007, and ηp^2^ = 0.053) and Zoo Map 1 [*F*(1,31) = 5.72, *p* = 0.018, and ηp^2^ = 0.042], and a medium-large effect size for the Six Parts test [*F*(6,126) = 4.39, *p* < 0.001, and ηp^2^ = 0.173]. Table 6 and Figure 2 provides a summary of these comparisons.

**TABLE 6:** BADS-C subtest scores according to SDQ categorization, and the statistical comparisons.

	SDQ	Mean	SD	Comparison	Significance	Effect size
Playing cards	Low	10.01	2.672	*F*(1,31) = 0.011	*p* = 0.916	ηp^2^ = 0.000
	High	9.90	3.023			
Water	Low	10.28	3.216	*F*(1,31) = 0.964	*p* = 0.328	ηp^2^ = 0.007
	High	9.71	2.700			
Key search	Low	12.50	3.460	*F*(1,31) = 7.389	*p* = 0.007	ηp^2^ = 0.053
	High	10.76	3.001			(small-med)
Zoo map 1	Low	10.82	2.687	*F*(1,31) = 5.716	*p* = 0.018	ηp^2^ = 0.042
	High	9.55	3.039			(small-med)
Zoo map 2	Low	10.32	2.726	*F*(1,31) = 0.721	*p* = 0.398	ηp^2^ = 0.005
	High	10.05	3.164			
Six part	Low	10.89	2.861	*F*(1,31) = 13.391	*p* = 0.001	ηp^2^ = 0.093
	High	9.02	3.005			(med-large)
CBADS total	Low	64.82	7.78	*F*(6,126) = 4.39	*p* < 0.001	ηp^2^ = 0.173
	High	58.98	9.19			(large)

The equivalent analysis was also conducted on the factor scores of the DEX-C (abstraction, monitoring, and six parts). The multivariate effect was significant [*F*(3,129) = 6.15, *p* = 0.001, and ηp^2^ = 0.125], as were the between subjects tests of the factor scores, with a medium-large effect for six elements [*F*(1,131) = 13.39, *p* < 0.001, and ηp^2^ = 0.093] and small-medium effect for both monitoring [*F*(1,131) = 6.171, *p* < 0.011, and ηp^2^ = 0.04] and abstraction [*F*(1,131) = 4.20, *p* < 0.042, and ηp^2^ = 0.031] scores. There was also statistically significant difference between the two groups on DEX total scores and factor scores but as this is expected given the concordance in response format and overlap in some items of the two questionnaires the results are not presented in full.

## Discussion

In summary, this study observed that the BADS-C has a two factor structure comprising monitoring and abstraction processes. The monitoring factor loads most strongly on Playing Cards and Zoo Map tasks, whereas the abstraction factor loaded on the Key Search and Water tasks. The Six Parts task did not load strongly on either factor. Secondly, the DEX-C questionnaire has a three-factor structure considered to represent behavioral and cognitive expressions of the dysexecutive syndrome, along with a weaker factor associated with aspects of social and emotional responsiveness. Thirdly, a consistent pattern of low-moderate correlations between BADS-C and DEX-C scores and SDQ scores, an established and widely used measure in children and young people was observed. Finally, a significant difference in the BADS-C performance of children and adolescents from the general population categorized as low (≤25th percentile) or high (≥75th percentile) on the SDQ total difficulties scale, even when the estimated IQ difference between the two groups is notable. This observation holds for the BADS-C total score as well as several individual subtests.

On the basis of these results, it seems reasonable to conclude that BADS-C is a valid EF measure among children and adolescents aged between 8 and 16 years. Given the limited evidence of psychometric validity of EF measures in widespread use (Nyongesa et al., 2019), this observation is of particular note. In addition, factor analysis indicates that the measures map onto established theoretical conceptualizations of the executive functions, whereas the third and fourth main findings indicate that the measure is sensitive to everyday difficulties that can be experienced by children with executive dysfunction (i.e., problems with conduct, hyperactivity, peer relationships, and emotional experience and regulation). Notwithstanding the regression models based upon BADS-C scores (either total score, or those derived from the factor analysis) were significant, they only accounted for a small amount (<10%) of the variance in everyday functioning. Though this means that BADS-C scores *alone* are unlikely to be useful in predicting the occurrence of everyday difficulties, this finding must be interpreted in the light of the following considerations. Firstly, the SDQ, though the best available screening measure for these purposes, addresses a broad range of domains, and as such includes many symptoms that would not be expected to relate to executive functions (e.g., particularly emotional subscale items such as “often complains of headaches, stomach-aches or sickness,” and “many fears, easily scared”). Secondly, there are obviously many biological, social and psychological factors that influence the expression of problems in the SDQ domains. Viewing the findings from this broad context, that performance on a “snapshot” test of EF accounts for *any* variance in reported everyday problems in a representative normative sample is noteworthy. This finding is supported by the subsequent analysis of children scoring in the upper and lower quartiles of the SDQ total difficulties scale.

The factor structures observed to underlie the BADS-C and DEX-C make intuitive sense in their separation between abstraction/monitoring and cognitive/behavioral aspects of executive level problems, respectively. Whilst they do not map completely onto any one theoretical model, the factors are broadly consistent with Stuss and Benson’s (1984) description of deficits that arising from frontal lobe damage. Given the aims of the development of the BADS and DEX were to develop an ecologically valid and sensitive test of EF, this is an expected pattern of results. The DEX-C’s factor structure deviated from that reported by Burgess et al. (1998) in relation to the DEX, who identified five factors namely inhibition, intentionality, executive memory, positive affect, and negative affect. However, Burgess et al. (1998) reported that the DEX was designed with four domains in mind, specifically emotion/personality, motivation, behavior and cognition. Whilst there is no straightforward correspondence with the currently identified structure, there is certainly a large degree of overlap. The discrepancies in the derived factor structure between the child and adult versions of the questionnaire may result from differences in the presentations of dysexecutive syndrome in adults versus children, or difference in the nature of executive “symptoms” in children from the general population rather than children presenting clinically with executive dysfunction.

The scores obtained from the factor analyses explained a higher proportion of the variance in SDQ total difficulties than the total scaled score, but this increase was not sufficient to warrant the development of a revised scoring system for the BADS-C. It is also unlikely that the scores could be used to predict the likelihood of everyday difficulties on an individual basis. However, the identification of this factor structure could aid the interpretation of obtained test results. If these factors are kept in mind when examining the profile of a child’s scores, then this provides additional information upon which recommendations for rehabilitation and/or management strategies may be based. For example, structured problem-solving techniques could be useful for children with difficulties on tasks that make up the abstraction component, whereas time management strategies, checklists, reminders, and cueing devices might be of more value for children displaying difficulties on tests that tap into the monitoring factor. These findings add further support to use of the BADS-C in populations likely to present with executive level difficulties as exemplified in recent studies involving BADS-C in Portuguese children with ADHD (de Almeida et al., 2014), French young people with frontal lobe tumors (Longaud-Vales et al., 2016) as well as Italian children with Neurofibromatosis Type I (Riva et al., 2017) to name but some. In conclusion, the evidence presented here suggests that the BADS-C and DEX-C are valid EF measures in children and adolescents which chart age-related developmental trajectories and as such may be of utility in academic and clinical pediatric neuropsychology practice.

## Data Availability Statement

The original contributions presented in the study are included in the article/supplementary material, further inquiries can be directed to the corresponding author/s.

## Ethics Statement

The studies involving human participants were reviewed and approved by NHS Cambridge Local Research Ethics Committee (LREC) Ethics Committee. Written informed consent to participate in this study was provided by the participants’ legal guardian/next of kin.

## Author Contributions

JF: undertook a secondary analysis of test development data. JF and FW: contributed to the manuscript text equally. Both authors contributed to the article and approved the submitted version.

## Conflict of Interest

The authors declare that the research was conducted in the absence of any commercial or financial relationships that could be construed as a potential conflict of interest.

## Footnotes

1 Though the majority of age-scaled scores are also adjusted for approximate IQ, Key Search and Zoo Map 2 do not, hence IQ as a co-variate in this analysis.
